# Innervation of the hip joint capsular complex: A systematic review of histological and immunohistochemical studies and their clinical implications for contemporary treatment strategies in total hip arthroplasty

**DOI:** 10.1371/journal.pone.0229128

**Published:** 2020-02-26

**Authors:** Joanna Tomlinson, Johann Zwirner, Benjamin Ondruschka, Torsten Prietzel, Niels Hammer

**Affiliations:** 1 Department of Anatomy, School of Biomedical Sciences, University of Otago, Dunedin, Otago, New Zealand; 2 Institute of Legal Medicine, Faculty of Medicine, University of Leipzig, Leipzig, Saxony, Germany; 3 Department of Orthopaedics, Trauma and Reconstructive Surgery, Zeisigwaldkliniken Bethanien, Chemnitz, Saxony, Germany; 4 Department of Orthopaedic, Trauma and Plastic Surgery, University of Leipzig, Leipzig, Saxony, Germany; 5 Division of Medical Technology, Fraunhofer Institute for Machine Tools and Forming Technology (Fraunhofer IWU), Dresden, Saxony, Germany; 6 Institute of Macroscopic and Clinical Anatomy, University of Graz, Graz, Styria, Austria; University of Memphis, UNITED STATES

## Abstract

The hip joint capsule contributes to the stability of the hip joint and lower extremity, yet this structure is incised and often removed during total hip arthroplasty (THA). Increasing incidence of osteoarthritis is accompanied by a dramatic rise in THAs over the last few decades. Consequently, to improve this treatment, THA with capsular repair has evolved. This partial restoration of physiological hip stability has resulted in a substantial reduction in post-operative dislocation rates compared to conventional THA without capsular repair. A further reason for the success of this procedure is thought to be the preservation of the innervation of the capsule. A systematic review of studies investigating the innervation of the hip joint capsular complex and pseudocapsule with histological techniques was performed, as this is not well established. The literature was sought from databases Amed, Embase and Medline via OVID, PubMed, ScienceDirect, Scopus and Web of Science; excluding articles without a histological component and those involving animals. A total of 21 articles on the topic were identified. The literature indicates two primary outcomes and potential clinical implications of the innervation of the capsule. Firstly, a role in the mechanics of the hip joint, as mechanoreceptors may be present in the capsule. However, the nomenclature used to describe the distribution of the innervation is inconsistent. Furthermore, the current literature is unable to reliably confirm the proprioceptive role of the capsule, as no immunohistochemical study to date has reported type I-III mechanoreceptors in the capsule. Secondly, the capsule may play a role in pain perception, as the density of innervation appears to be altered in painful individuals. Also, increasing age may indicate requirements for different strategies to surgically manage the hip capsule. However, this requires further study, as well as the role of innervation according to sex, specific pathology and other morphometric variables. Increased understanding may highlight the requirement for capsular repair following THA, how this technique may be developed and the contribution of the capsule to joint function and stability.

## Introduction

Increasing numbers of total hip arthroplasties (THA) are performed each year globally [1, 2]. This procedure is noted to be highly successful. However, around 7–17% of THAs across the world between 2001 and 2016 required surgical revision for various reasons [1–3]. Dislocation of the hip joint is one of the most common reasons for operative revision within the first year [3, 4]. The capsular complex of the hip joint is important for maintaining stability of the hip joint [5], comprising of the iliofemoral, ischiofemoral, pubofemoral and zona orbicularis ligaments and joint capsule. There is debate whether preserving and repairing the capsule during THA is advantageous or not [6], yet the literature supports that capsular repair during THA has better outcomes, with lower dislocations and revision rates compared to primary THA without repair [6–16]. This may be a result of partial restoration of physiological joint stability through preservation and repair of the capsule, consequently reducing the risk of further dislocation. However, it is important to note that the success of THA also is subject to numerous variables [7, 12, 17, 18], including the surgical approach, implant material and type, femoral head size, cup inclination and history of neurological and/or vascular disability and disease.

Emerging research investigates the biomechanical role and properties of the capsule [19–23]. This indicates that the contribution of the capsule to the mechanics of the joint may be more extensive than passive stabilization alone, particularly as no significant difference has been noted between the biomechanical properties of the capsular ligaments [22]. This is despite research indicating that the posterior approach to the capsule is at greater risk of post-operative dislocation when repaired or excised [24, 25]. Although the risk may be higher due to excising the capsule in the most common dislocating direction, which is a result of injury mechanisms and differences in bony or muscular architectural support to the joint. This risk is reduced by using native size femoral head implants [24], which indicates that numerous factors contribute to maintaining stability. Ligamentous receptors in the capsule may also work alongside muscular spindles and cutaneous receptors to aid in the active stabilisation of the hip joint, through contribution to neuromuscular feedback of the joint. The contribution of each of these to joint proprioception and stability is yet to be clarified in the hip joint [26, 27]. Recent research proposed that the capsule may have a proprioceptive role as indicated by the presence of mechanoreceptors [28]. These mechanoreceptors (Ruffini, Pacinian, Golgi-like corpuscles and free nerve endings [FNEs]) were first classified by Freeman and Wyke in 1967 [29]; then this classification was subsequently modified for application to humans [30, 31]. Each of these are thought to play a role in joint proprioception, by monitoring different parameters of joint position and movement.

The relationship of mechanoreceptors with proprioceptive function has not been studied in the human hip capsule, to date. However, this relationship may be inferred from mechanoreceptor density, as a positive correlation with proprioceptive function has been noted in the ligaments of the knee [32, 33]. This further highlights the opportunity to gain a more detailed understanding about the contribution of the capsule to proprioception of the hip joint. This also provides an opportunity to gain increased understanding about the changes that occur in post-operative tissue, for example following THA with and without closure of the capsule. If the capsule is preserved and repaired, this is described as being healed capsular tissue. The post-operative scar tissue, replacing the excised capsule, is called as the pseudocapsule. This is characterised as a crude fibrous envelope with hyalinization [34] as a consequence of the suturing, which is shown to take up to 6–8 weeks to mature into a tight fibrous pattern to prevent leakage of synovial fluid and dislocation [35]. Gaining increased understanding may also aid in determining if THA with repair of the capsule is crucial and how it may be developed further. This review therefore aims to encompass the histological studies on innervation of the human native hip joint capsular complex. In addition, it discusses the post-operative innervation in the pseudocapsule and the changes in these structures in healthy individuals, those with pathology or according to demographic parameters—none of which have been reviewed to date [36]. As well as changes in density and distribution across the capsular complex and if these are consistent with macroscopic findings [36]. Increased understanding of the innervation of the capsule could determine if this is potentially causative for successful THA. In addition, it may highlight areas for development, such as any alterations to surgical management, limitations of surgical incisions, areas to avoid and changes required based on demographic and anthropometric variables.

## Methods

A systematic review of the literature available from approximately 1900 was performed in order to identify peer-reviewed articles according to the PRISMA (Preferred Reporting Items for Systematic Reviews and Meta-analyses) guidelines [37]. Keywords relating to the innervation of the hip joint capsular complex and pseudocapsule in histological studies were searched using the following online databases: Amed, Embase and Medline via OVID, PubMed, ScienceDirect, Scopus and Web of Science. Article selection was performed manually by one author (JT) employing database configuration where possible to identify original research papers that used histological methodologies to investigate the innervation of the capsular complex or pseudocapsule. Papers were excluded from inclusion in this review based on their titles, then their abstract and then the full paper was assessed. The exclusion criteria were: only the abstract or title were available, as this does not allow the paper to be critically analysed by the authors of this review. Animal studies were also excluded, as quadruped anatomy is potentially not comparable to bipedal human morphology. Studies were excluded if they did not include a histological component to their research, nor study the hip nor innervation. The full text of the articles that met these criteria were obtained and then a manual backwards search was performed by screening all cited references within these papers, in order to identify additionally relevant papers. A forward search was undertaken using the Web of Science Core Collection. The papers identified through this forward and backwards chain sampling were also subject to the same exclusion criteria.

The quality of these articles was assessed by one author (JT) to determine the risk of bias. A checklist which was modified from recommendations for histological studies [38] and checklists for observational studies [39] was used, as no suitable checklist is available for the observational histological studies. One point was awarded for each question on the components of the study that was answered with a yes, to a maximum of eleven. A score of 0–4 was deemed as low quality, a score of 5–8 as moderate quality and 9–11 as high quality. Greater emphasis was placed on studies with high quality in the synthesis of the literature.

Data was extracted from the articles by one author (JT) and rechecked by the same author. Analysis and synthesis of studies was performed by one author (JT) to assess the morphology, density, distribution of mechanoreceptors, free nerve endings and nerve fibres in the capsular complex and pseudocapsule. The changes in innervation according to demographic variables (age, sex and ethnicity), anthropometric variables (height, weight, lower limb and pelvic dimensions) and pathology were also assessed. Papers written in languages other than English were translated by native or fluent speakers, before performing data extraction.

## Results

### Selection of studies

**Fig 1:**
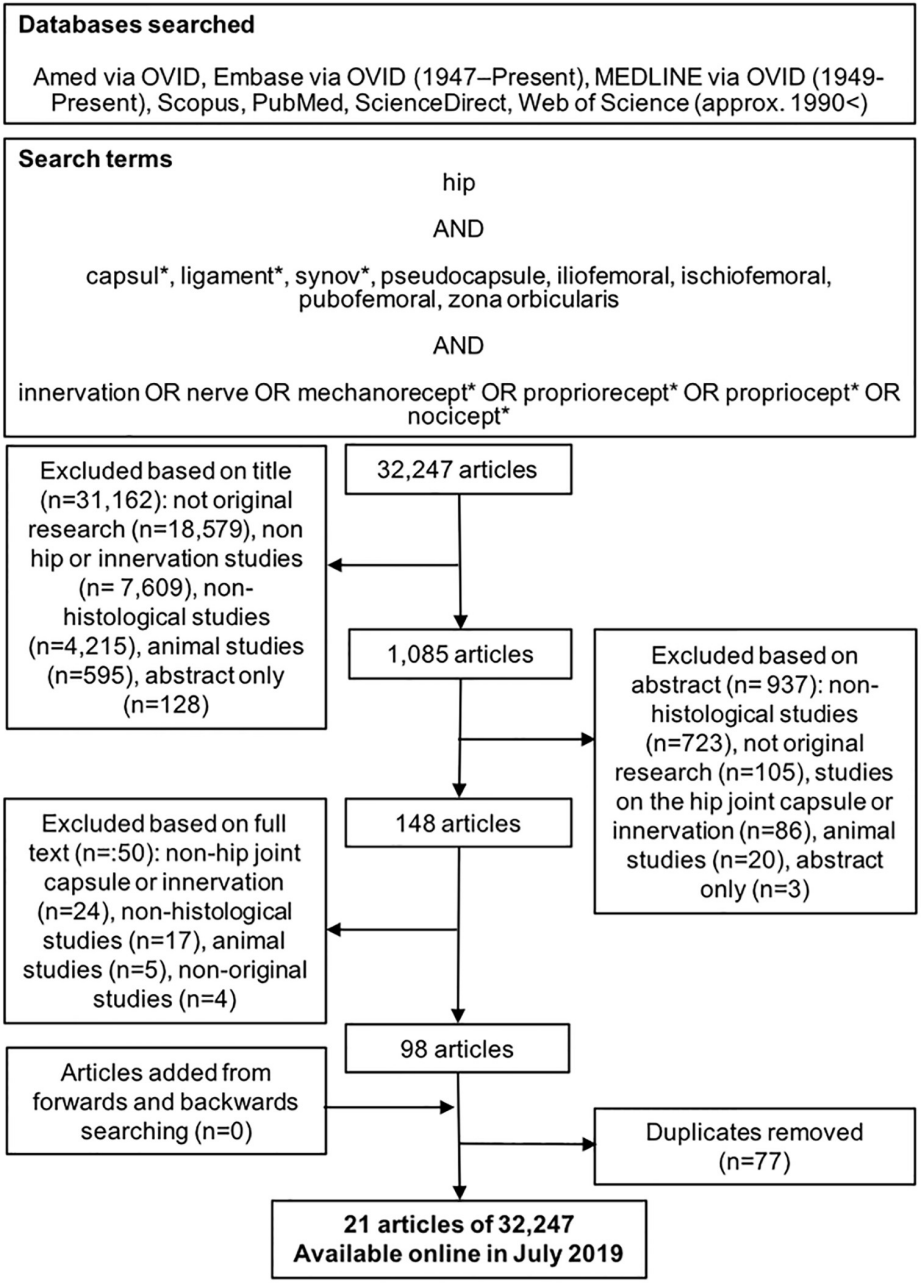
PRISMA flowchart of systematic review process. Systematic review process, including databases, keywords, number of papers retrieved, inclusion and exclusion criteria and results from backwards and forwards searching [37].

To date, little research investigates the distribution of innervation patterns across the capsular complex of the hip joint, the healed capsular tissue or the pseudocapsule following surgical intervention (Fig 1). Following an encompassing search of the published literature on the topic, which was completed in July 2019, 32,247 articles were identified. Finally following screening of articles, 21 articles were selected for analysis and their risk of bias was assessed—as shown in Table 1. Two were deemed as low quality/ high risk of bias, sixteen were moderate quality and three were high quality/ low risk of bias. No papers were excluded based on their quality, as they all appeared to contain important information on the innervation of the hip capsule. Papers selected were predominantly written in English (n = 17/21), with two in Russian [40, 41], one in German [42] and another in Japanese [43]. These papers were translated into English by one of our multi-lingual authors and other colleagues who are native or fluent speakers. The papers included were published between 1964 and 2019.

**Table 1:** Quality analysis of papers included in this review, using a modified quality assessment tool for histological observational studies, adapted from Knijn et al. (2015) and Manterola and Otzen (2017) [38, 39].

Papers	Level of evidence	Is the hypothesis/ aim clear?	Does it indicate the number of samples studied?	Sample characteristics clearly described?	Are confounding variables between/within groups described?	Is the method for tissue retrieval clearly described?	Is the applied histological protocol clearly described?	Were methods to reduce bias described?	Does the study specify the statistical method employed?	Are the results presented clearly?	Are limitations and existing risk of bias described?	Is there a conclusion?	Total score
**Li et al (2019) [44]**	4	Y	Y	Y	Y	N	Y	N	Y	Y	N	Y	**8**
**Vasconcelos et al. (2016) [45]**	4	N	Y	Y	Y	N	Y	N	Y	Y	Y	Y	**8**
**Naim Syed et al. (2014) [34]**	4	Y	Y	Y	Y	N	N	Y	N	N	Y	Y	**7**
**Desteli et al. (2014) [46]**	4	Y	Y	N	N	Y	Y	Y	Y	Y	Y	Y	**9**
**Grzegorzewski et al. (2014) [47]**	4	N	Y	Y	Y	N	Y	N	Y	Y	N	Y	**7**
**Haversath et al. (2013) [48]**	4	N	Y	N	Y	Y	N	Y	Y	N	Y	Y	**7**
**Gerhardt et. (2012) [49]**	4	Y	Y	Y	N	Y	Y	N	N	Y	N	Y	**7**
**Takeshita et al. (2012) [50]**	4	N	Y	Y	Y	N	Y	Y	Y	Y	Y	Y	**9**
**Moraes et al. (2011) [28]**	4	Y	Y	Y	N	Y	Y	N	Y	Y	N	Y	**8**
**Maslon et al. (2011) [51]**	4	N	Y	Y	N	Y	Y	N	Y	Y	N	Y	**7**
**Lehner et al. (2008) [52]**	4	Y	Y	N	Y	N	Y	Y	Y	Y	N	Y	**8**
**Saxler et al. (2007) [53]**	4	Y	Y	N	N	Y	Y	N	Y	Y	N	Y	**7**
**Saxler et al. (2005) [42]**	4	N	Y	Y	N	Y	Y	N	N	Y	N	Y	**6**
**Gáspár et al. (2004) [54]**	4	N	Y	N	N	N	Y	N	Y	Y	Y	Y	**6**
**Muratli et al. (2004) [55]**	4	Y	Y	Y	Y	Y	N	Y	N	Y	Y	Y	**9**
**Niissalo et al. (2002) [56]**	4	Y	Y	N	Y	N	Y	N	N	Y	N	N	**5**
**Bosetti et al (2001) [57]**	4	Y	Y	Y	Y	Y	Y	Y	N	Y	N	N	**8**
**Rabinowicz and Jacqueline (1990) [58]**	4	Y	Y	Y	Y	N	Y	N	N	Y	N	N	**6**
**Golub and Bronovitskaia (1981) [40]**	4	N	N	N	N	N	N	N	N	N	N	N	**0**
**Nettov and Iankovskaia (1978) [41]**	4	N	Y	Y	N	N	N	N	N	N	N	N	**2**
**Hosokawa (1964) [43]**	4	N	Y	N	N	Y	Y	N	N	Y	N	Y	**5**

Studies are listed in chronologically descending order and assessed to measure the risk of bias and quality of the study. One point was awarded for each question on the components of the study that was answered with a yes, to a maximum of eleven.

### Demographic and anthropometric data

Samples included in the current literature varied according to the size studied, and within this by age, sex, pathology, and disease progression, as shown in Table 2. Individuals covered a broad age range, including intrauterine fetuses, from an undefined age to 93 years old. Anthropometric variables (weight and height) were included in one study [52]. This study investigated the hip capsular complex in one group of individuals; therefore, no inferences could be made from weight and height of these individuals as no comparisons were made. No other study investigated anthropometric variables such as height, weight, body mass index, pelvic or lower limb dimensions. No other demographic or anthropometric variables were studied in the included literature.

**Table 2:** Demographic data of included groups from studies identified from the literature review.

Author	Sample size	Sex ratio (male: female)	Age range	Tissue studied	Pathology studied
**Li et al (2019) [44]**	80 (G1- 45 G2- 35	G1- 7:38 G2- 8:27	G1- NS. Mean 27 years G2- NS. Mean 30 years	Synovial membrane	G1- Moderate DDH and OA G2- Severe DDH and OA
**Vasconcelos et al. (2016) [45]**	15	6:9	37–83 years	Synovial membrane	OA
**Naim Syed et al. (2014) [34]**	20	NS	NS. Mean 76 years	Pseudocapsule	OA revision THA
**Desteli et al. (2014) [46]**	30 (G1- 15 G2-15)	NS	G1- Preterm fetus G2- 6–18 months	Capsule	G1- Fetus with NKP G2- DDH
**Grzegorzewski et al. (2014) [47]**	34 (G1- 19 G2-15)	G1- 10:9 G2- 4–11	G1- 5–18 years G2- 2–4 years	Capsule	G1- CP and hip dislocation G2- DDH and hip dislocation
**Haversath et al. (2013) [48]**	57 patients (G1-34)	29:28 G1-NS Other- NS	8–87 years G1- NS Other- NS	G1- Capsule Other- Labrum, LHF	AVN, CO, FAI, OA, SHD G1- NS Other- NS
**Gerhardt et al. (2012) [49]**	8	5:3	68–93 years	Capsule	Mild to moderate OA (n = 5), severe OA (n = 3)
**Takeshita et al. (2012) [50]**	62 G1- 50 G2-12	G1- 4: 46 G2- 3:9	G1- 48–80 years G2- 63–90 years	Capsule	G1- OA G2- FNF
**Moraes et al. (2011) [28]**	45 (G1- 30 G2-15)	45:0	G1- 38–75 years G2- 21–50 years	Capsule, labrum, LHF	G1- OA G2- NKP
**Maslon et al. (2011) [51]**	19	10:9	5–18 years	Capsule	CP and hip dislocation
**Lehner et al. (2008) [52]**	15	7:8	NS. Mean 67 years	Capsule and synovial membrane	Hip OA
**Saxler et al. (2007) [53]**	9 (G1- 3 G2- 3 G3- 3)	G1- 0:3 G2- NS G3- 0:3	G1- 74–75 years G2- 55–78 years G3- 52–80 years	G1- Capsule G2- Pseudocapsule G3- Capsule	G1- OA G2- pain free THA G3- FNF
**Saxler et al. (2005) [42]**	6 (G1- 3 G2- 3)	0:6	G1- 74–75 years G2- 52–80 years	Capsule	G1- OA G2- FNF
**Gáspár et al. (2004) [54]**	22	NS	NS	Capsule and synovial membrane	OA
**Muratli et al. (2004) [55]**	20	12:8	6–20 months	Capsule	DDH
**Niissalo et al. (2002) [56]**	15 (G1- 4 G2- 11)	G1- 2:2 G2- 9:2	G1-1 66–75 years G2- 37–88 years	G1- Pseudocapsule G2- Capsule	G1-OA revision THA G2- OA, ON
**Bosetti et al (2001) [57]**	8	NS	NS. Mean 68 years	Pseudomembrane and capsule	AL
**Rabinowicz and Jacqueline (1990) [58]**	52 (46 patients G1- 5 G2-13 G3- 10 G4- 12 G5- 6)	G1- 5:0 G2- 12:1 G3- 10:0 G4- 6:6 G5- 1:5	G1- 30–73 G2- 56–90 G3- 37–59 G4- 40–77 G5- 48–64	Capsule and synovial membrane	G1- AS G2- FNF G3- IO G4- OA G5- RA
**Golub and Bronovitskaia (1981) [40]**	NS	NS	Fetus	Capsule	NS
**Nettov and Iankovskaia (1978) [41]**	56	34:22	30–55 years	Capsule	OA
**Hosokawa (1964) [43]**	2	NS	NS	Capsule	NKP

Studies are listed in chronologically descending order. Groups are labelled as G1, G2, etc. The abbreviations are related to the following pathologies, AL = aseptic loosening, AS = ankylosing spondylitis, AVN = avascular necrosis of the femoral head, CO = corrective osteotomy, CP = cerebral palsy, DDH = developmental dysplasia of the hip, FAI = femoroacetabular impingement, FNF = femoral neck fracture, IO = idiopathic osteonecrosis, LHF = ligament of the head of the femur, NKP = no known pathology, NS = not stated, OA = osteoarthritis, ON = osteonecrosis of femoral head, RA = rheumatoid arthritis, THA = total hip arthroplasty. Some studies investigated samples that were not relevant to the aims of this review, therefore they were not included in the results.

The demographics of samples varied considerably between the studies incorporated in this review. Three studies investigated the innervation of the capsular complex in individuals with no known pathology [28, 43, 46]. One study did not state if the specimens were healthy or pathological [40]. The other 17 studies only investigated the capsular complex or pseudocapsule with diverse pathology; these are displayed in Table 2. No study investigated the innervation of the healed capsular tissue. Seven studies compared findings from hip OA patients with other groups. The severity of the clinical diagnosis was recorded in eight studies. Some researchers define this according to diagnostic criteria of the joint the tissue was collected from [28, 44, 45, 50, 55]. These include the use of Bombelli’s classification [28, 59], Crowe classification [44, 60], the Harris hip score [50, 61], the Kellgren and Lawrence classification (which grades OA severity according to radiological findings) [44, 50, 62], and Tönnis classification [45, 55, 63]. This therefore allows for a small degree of comparison between studies. Others stated broad definitions for disease progression [49, 54, 58], such as “mild to moderate degenerative osteoarthritic changes” [49]. This highlights the numerous individual variables present between the samples studied in the current literature.

### Methodological data

The literature presents a variety of methodologies to investigate the innervation of the capsular complex. In particular, the tissue origin and preparation varied considerably; this is outlined in Table 3. Only 13 studies reported the thickness of the section which ranged from 3–60 μm [28, 42, 44–46, 48–50, 52–54, 56, 57].

**Table 3:** Table representing tissue origin, basic preparation and storage represented in the literature.

	Tissue from surgery	Tissue from cadavers	Decalcified	Paraffin embedded	Frozen tissue
**Studies**	**18**	**3**	**2**	**9 of 18**	**9 of 18**
**Specimens**	**474**	**25**	**60**	**286**	**250**

Fifteen studies stained tissue with immunohistochemical antibodies and histology stains, all of which used specimens obtained from surgery. Another four studies used only conventional histological methods [28, 43, 49, 58] and two studies did not disclose the stain used [40, 45]. Various histological stains were employed in these four studies: gold chloride staining [28, 49], Bielschowsky-Seto silver [43], haematoxylin and eosin (H&E), van Gieson [58], Luxol fast blue, silver impregnation of Glees, of Tibor-pap and of Gomori.

In the immunohistochemical studies, not all studies described their methodology extensively. Three studies stated the protocol employed to unmask antigens [44–46]. Other studies did not disclose this information and therefore may have omitted this step. Many immunological markers were employed; these are displayed in Table 4. Each of these interact with different proteins, and therefore provide different results and insights of nerve expression and development.

**Table 4:** Table representing details of the immunological markers for nerve tissue used in the current literature.

Immunological markers	Antibody details
**CGRP**	Chemicon International Inc., Temecula, CA, USA [50]
	Rabbit antibody, 1:2000, Peninsula [53, 54]
	Rabbit antibody, 1:4000, Cambridge Research Biochemicals, Cambridge, UK [56]
	1:4000, Sigma Aldrich, St Louis, Missouri, USA [45]
	Not stated [44]
**GAP**	Mouse monoclonal GAP-43/B-50, Chemicom International Inc., Temecula, CA, USA [56]
**NF**	NF200, 1:1000, Abcam Inc, Cambridge, Massachusetts, USA [45]
	NF200 [44]
	Rabbit monoclonal 1:300, Novus Biologicals, Littleton, Colorado, USA [48]
	Not stated [54]
**NF-kß**	Nuclear factor kß p65, Santa Cruz Biotechnology, Santa Cruz, CA, USA [50]
**NK1**	Anti NK1 antibody, details not specified [42]
	Neurokinin 1, Sigma Aldrich, St Louis, Missouri, USA [54]
**Nociceptin**	Rabbit polyclonal 1:500, Acris Antibodies GmbH, Germany [48]
**NY**	Mouse polyclonal 1:1000, Abcam Inc. Cambridge, Massachusetts, USA [48]
	Rabbit C-flanking peptide of neuropeptide Y antibody, 1:4000, Cambridge Research Biochemicals, Cambridge, UK [56]
**PGP 9.5**	Rabbit antibody, 1:6000, Cambridge Research Biochemicals, Cambridge, UK [56]
**S100**	Monoclonal antibody [34]
	Mouse monoclonal, Neomarkers, Fremont, California, USA [55]
	Mouse monoclonal, 1:25, Novocastra, UK [47, 51]
	Rabbit monoclonal, 1:100, Acris Antibodies GmbH, Herford, Germany [48]
	Rabbit anti-human RB9018P, Thermo Fisher Scientific, Waltham, MA, USA [46]
	Polyclonal antibody, Immunon, Pittsburgh, USA [57]
**SP**	Rabbit antibody, 1:4000, Cambridge Research Biochemicals, Cambridge, UK [56]
	Rabbit monoclonal, 1:500, Acris Antibodies GmbH, Herford, Germany [48]
	Rabbit polyclonal, 1:2000, Chemicon International Inc., Temecula, CA, USA [47, 51]
	Rabbit polyclonal, Ab1566, Chemicon International Inc., Temecula, CA, USA [52]
	Rabbit Substance P, 1:500, ICN [42, 53]
	Substance P, INSTAR [54]
	Not stated [44]
**TH**	Anti—tyrosine hydroxylase, rabbit polyclonal, Ab152, Chemicon International Inc., Temecula, CA, USA [52]
	Anti–tyrosine hydroxylase, 1:100, Millipore, Burlington, Massachusetts, USA [45]
**TuJ-1**	Neuron specific class III ß-tubulin, Convance, Princeton, NJ, USA [50]
**VIP**	Rabbit antibody, 1:10,000, Cambridge Research Biochemicals, Cambridge, UK [56]

Several immunological markers were used to attempt to stain nerves and mechanoreceptors in the hip capsule, synovial membrane and the post-operative pseudocapsule. The full details of the antibodies were not stated in all papers. General nerve markers are S100, PGP 9.5, NF and TuJ-1. S100: Soluble protein 100, is noted to stain Schwann cells, NF = neurofilament, PGP 9.5 = protein gene product 9.5, TuJ-1 = Neuron specific tubulin stain the cell axon. Sensory nerve markers are SP, CGRP, NK1 and nociceptive. SP = substance P, CGRP = calcium gene-related peptide, nociception, NK1 = neurokinin 1. Sympathetic nerve markers are NY = Neuropeptide Y, TH = Tyrosine hydroxylase, VIP = vasoactive intestinal polypeptide. Nerve growth is marked by GAP. GAP: growth associated protein. Cellular response to painful stimuli is marked by NF-kß = nuclear factor kß.

### Mechanoreceptors

#### Presence of mechanoreceptors

The presence of mechanoreceptors has been studied in capsular tissue with no known pathology [28, 43, 46], yet these structures have only been found in two of three studies [28, 43]. One of these studies included an adult cohort [28] and the other did not disclose the age of the individuals that samples were obtained from [43]. Mechanoreceptors have not been described in foetuses using immunohistochemical techniques [46]. However, some research has indicated that nerve endings may develop in the capsule before the fetus is 9cm [40] (around 14 weeks in utero), yet the description of the development is unclear and this study has a high risk of bias as the methodology to mark these endings is not stated.

Ruffini, Pacinian and Golgi tendon organ mechanoreceptors have been reported in the hip joint capsular tissue of individuals with OA and other pathologies [28, 49, 58]. Some of this research did not specify which individuals expressed mechanoreceptors, thus preventing comparison between pathologies [58].

Mechanoreceptors have only been noted using conventional histology techniques to date. Most commonly these have been found using gold chloride staining [28, 49]. In contrast, when using immunological markers mechanoreceptors appear to be absent in the hip capsules of patients with developmental dysplasia of the hip (DDH) [46, 47, 55], cerebral palsy [47, 51], and in the post-operative pseudocapsule of those undergoing revision of their THA [34], which may indicate true absence or errors in methodologies. Mechanoreceptors appear to be present in the hip capsule; however, to date their presence has not been proven with immunohistochemical markers. This information is summarised in Table 5.

**Table 5:** Comparison of studies representing the immunohistochemical marker types, tissue type and nerve tissue structures noted in literature.

Papers	Sample—Disease	General nerve marker		Sensory nerve marker		Sympathetic nerve marker	
**Li et al (2019) [44]**	DDH and OA	nf		nf			
**Vasconcelos et al. (2016) [45]**	OA	nf		nf		nf	
**Desteli et al. (2014) [46]**	DDH	FNE					
**Desteli et al. (2014) [46]**	NKP	FNE					
**Naim Syed et al. (2014) [34]**	2 THA	FNE					
**Grzegorzewski et al. (2014) [47]**	CP	nf		nf			
**Grzegorzewski et al. (2014) [47]**	DDH	nf		nf			
**Haversath et al. (2013) [48]**	1 THA	nf, FNE		nf, FNE		nf, FNE	
**Takeshita et al. (2012) [50]**	FNF	None noted		None noted			
**Takeshita et al. (2012) [50]**	OA	nf		nf			
**Maslon et al. (2011) [51]**	CP	nf, FNE		nf, FNE			
**Lehner et al. (2008) [52]**	OA			N	nf	N	nf
**Saxler et al. (2007) [53]**	FNF			nf, FNE			
**Saxler et al. (2007) [53]**	OA			nf			
**Saxler et al. (2007) [53]**	Pain-free failed THA			None noted			
**Saxler et al. (2005) [42]**	OA			nf			
**Saxler et al. (2005) [42]**	FNF			nf			
**Muratli et al. (2004) [55]**	DDH	None noted					
**Gáspár et al. (2004) [54]**	OA	nf	nf	nf	nf		
**Niissalo et al. (2002) [56]**	OA 1 THA	nf		nf		nf	
**Niissalo et al. (2002) [56]**	OA 2 THA AL	nf, FNE		nf		nf	
**Bosetti et al (2001) [57]**	OA 2 THA AL	nf					
Key							
	Capsular ligament						
	Synovium						
	Pseudocapsule						

General nerve markers include S100, neurofilament protein, PGP 9.5 and neuron specific tubulin. Sensory nerve markers included are substance P, calcitonin gene-related peptide and nociceptin. Sympathetic nerve markers include neuropeptide Y, tyrosine hydroxylase, C-flanking peptide of neuropeptide Y and vasoactive intestinal polypeptide. FNE = free nerve ending, nf = nerve fibre, AL = aseptic loosening, CP = cerebral palsy, DDH = developmental dysplasia of the hip joint, FNF = fractured neck of femur, NKP = no known pathology, OA = osteoarthritis, THA = total hip replacement, the 1 and 2 refer to primary and secondary.

#### Distribution of mechanoreceptors

Few studies have described the distribution of mechanoreceptors across the capsule according to their anatomical location [43, 49]. In healthy individuals, the majority of mechanoreceptors were noted in the ligament, in comparison to the synovium [43]. Analysis of capsular innervation of each of the capsular ligaments indicated that a higher density of mechanoreceptors are present superior-laterally in the iliofemoral ligament in both healthy patients [43] and those with OA [49] and also at the insertion into bone. Fewer mechanoreceptors are noted inferiorly and posteriorly in the pubofemoral and ischiofemoral ligament [43, 49]. The distribution of each of the different types of mechanoreceptors has not been studied to date [43, 49].

One study noted the relationship of mechanoreceptors to other structures [43]. Golgi tendon organs and Krause corpuscles appear to be homogenously spread across the capsule with a potential spatial relationship to blood vessels in healthy subjects. This relationship has not been studied in capsules from individuals with hip pathology. In summary, the hip capsule appears to have greater innervation superior-laterally, however, the studies investigating distribution have a moderate risk of bias, use different methods to divide the capsule therefore these findings cannot be generalized to the greater population.

#### Structural changes of mechanoreceptors with pathology

There is general agreement that Pacinian, Ruffini and Golgi corpuscles are present in the hip capsule of individuals with no known pathology and patients with OA [28, 43]. However, this requires further clarification with regards to comparative density, location and structural proximities. One study noted that the morphology of mechanoreceptors in the capsule does not appear to differ between healthy individuals and those with OA [28]; however; others note destructive changes occur in mechanoreceptors in association to the progression of OA, causing damage to the mechanoreceptor’s capsule [41]. Qualitative analysis of the mechanoreceptors of the capsule and other soft tissue of the hip joint has been reported by one author, Moraes et al. (2011) [28] as follows:

- Ruffini corpuscles (type I mechanoreceptor [29]) had a diameter of around 100 μm and were described as globular ramifications.
- Pacinian corpuscles (type II mechanoreceptor [29]) had a diameter of around 50 to 100 μm, which were spherical in shape, and surrounded by external lamellas. Also, the similar Golgi-Mazzoni corpuscles have been noted in the hip capsule by Hosokawa [43].
- Golgi tendon organ or Golgi corpuscles (type III mechanoreceptor [29]) were noted to be up to 400 μm, and helical in shape.

Krause end bulbs, which monitor temperature [41, 43], encapsulated branched endings, encapsulated branched corpuscles and special corpuscles [43] have also been identified in human hip capsules; however, their morphology has not been reported. Unclassified corpuscles (type V mechanoreceptors [29]) have not been found in the hip capsule, to date. It is unclear whether the morphology of mechanoreceptors differs between healthy and arthrosis groups.

#### Effect of osteoarthritis on mechanoreceptor density

Osteoarthritis appears to reduce mechanoreceptor density, but to date no relationship has been noted with regards to demographic information, such as age or sex. The density of mechanoreceptors in the capsule has been quantified in patients with OA [28], but not any other pathology. The total number of mechanoreceptors was noted to be 0.044 per mm^2^ in those with OA [28] and 0.053 per mm^2^ in healthy individuals. However, these findings may not be applicable to all individuals, as this research only studied males and their findings combined the data from various soft tissues of the hip joint. In addition, the mean age of each of the groups differed by 21 years with 57 years for OA individuals and 36 years for non-OA cadavers. This therefore illustrates the many differing variables that occur simultaneously in the current literature.

A greater density of Pacinian and Ruffini corpuscles appear to be present in the hip capsules of both healthy individuals [28] and patients with OA [28, 49] compared to other mechanoreceptors. These mechanoreceptors act to monitor vibration and tensile loading, respectively. However, it is unclear if individuals have more Pacinian or Ruffini corpuscles in their hip capsular complex. When comparing the changes in density of specific mechanoreceptors in the hip capsules of those with and without OA Pacinian corpuscles are more greatly reduced in the OA group compared to the healthy group than the reduction in Ruffini corpuscles [28]. This may have an effect on biomechanical functioning of the joint, but more research is required to confirm this.

Overall, the literature has noted a few mechanoreceptors in the hip capsule across a small number of studies [28, 40, 43, 49, 58]; though they had limited sample sizes and also variance in the age and sex of samples studied. The current literature has been unable to note a relationship between mechanoreceptor distribution and anthropometric or demographic information. It is also unclear if mechanoreceptors are present in fetuses’ hip joint capsules [40, 46], whereas studies of healthy adults may have found mechanoreceptors [28], indicating that there may be changes in distribution according to age.

### Free nerve endings

#### Presence of free nerve endings

Consensus is present in studies using conventional histological and immunohistochemical markers that FNEs (type IV mechanoreceptors) are distributed across the hip capsule of healthy individuals [28, 43, 46], thus indicating the potential for a nociceptive role. FNEs have also been noted in those with OA [48], children with DDH [46, 47] and cerebral palsy [47, 51] with the immunohistochemical marker S100 alone [46] or in conjunction with other antibodies [47, 48, 51], as shown in Table 5. Despite these consistent findings, other authors have noted no FNEs with S100 alone in the hip capsules of those with DDH [55]. This may be a result of the use of an unsuitable stain, S100, which marks Schwann cells which are present as a thin layer or absent at the terminal portion of the free nerve ending. This suggests potential errors in these findings as a result of interpretation of FNEs. However, the absence of FNEs may also result from an irregular density of FNEs or alterations to FNE density that occur with pathology.

The presence of FNEs indicates the hip capsule plays a potential nociceptive role, as these are marked with sensory nerve markers: substance P, calcium gene-related peptide [53] and nociceptin [48]. However, other studies have been unable to report FNEs with the sensory marker NK1 [42], which interacts with substance P, indicating that these may be absent or may function to detect other modalities. The morphology of FNEs appears to be variable within healthy and OA patients [28, 43], but they generally do not appear to change between normal and OA groups [28]. The disparity in FNEs morphology may be a result of the presence of different subgroups of FNEs, including branched and unbranched nerve endings and unencapsulated glomerular endings [43]. This indicates that these FNEs may have different types of nociceptive functions. It is important to study these further in order to identify their morphology and functional significance.

#### Density of free nerve endings

The overall density of free nerve endings appears to be greater in healthy individuals compared to those with osteoarthritis [28], indicating potentially greater nociceptive functioning and response to painful stimuli. However, this difference may not have a functional significance, as the difference appears to be negligible between the groups, the groups were not matched according to age, and a significant difference was not assessed. The findings also lack validity as this study only investigated the anterolateral region of the capsule and combined their findings on the hip capsule with other soft tissue structures from the hip joint. Furthermore, these changes may be different in other regions.

Free nerve endings also appear to be more abundant than Golgi corpuscles, but less abundant than Pacinian and Ruffini corpuscles [28]. However, the importance of the nociceptive response of the capsule cannot be inferred from this information as it is unknown what region the FNEs or mechanoreceptors serve, and if the differences in proportions correlates to sensitivity to changes in biomechanical or nociceptive influences.

Several methodological discrepancies are present between studies investigating FNEs as they employ several different measures to quantify FNEs [28, 46] which limits the comparison of this information. There is also inconsistency in the thickness of the sections obtained in these studies, from 4 μm to 60 μm, which may result in different densities reported or an inability to visualise structures. However, it is important to note that nerve expression was reported in 60 μm sections, which may be FNEs or other mechanoreceptors [54].

Individual variation may account for discrepancies in current literature as different densities of FNEs have been reported in different pathologies. This includes no difference in FNEs density between healthy controls and DDH patients [46], no FNEs in the capsule of individuals with DDH [55], OA, fractured neck of femur, idiopathic necrosis or rheumatoid arthritis [58]. Changes in the density of FNEs may be due to errors in methodologies employed, due to variations in age, disease progression or other individual variation.

#### Distribution of free nerve endings

Literature is limited regarding description of the distribution of FNEs according to anatomical position in individuals with no known pathology [43], in patients with OA and other pathologies [48, 49]. In individuals with no known pathology the distribution or density of FNEs in comparison to other mechanoreceptors has not been defined [43]. However, research indicates that almost all nerve endings are present in the ligaments itself, compared to the synovium [43] and are mostly noted close to blood vessels [53], suggesting a functional relationship may be present, but this requires further study. In those with OA [49] and other pathologies [48] there is general agreement that the increased density of FNEs is present at the superior-lateral aspect compared to other regions of the capsule, the same region that more mechanoreceptors are thought to be located in healthy and individuals with OA [43, 49]. The inferior and posterior aspects of the capsule appear to lack FNEs, whereas they are evident in the anterior capsule [49], this is also consistent in healthy individuals [43].

Fewer FNEs appear to be present at the periphery of the capsule of individuals with various pathologies (AVN, CO, FAI, OA, SHD) compared to the medial and lateral aspects [48]. These findings are different to findings reported in subjects with no known pathology [43]. However, these studies may not be directly comparable as different regions of the capsule are investigated, resulting in the distribution of FNEs in the capsular complex still being unclear.

FNEs have also been noted in post-operative pseudocapsule tissue [34, 56], suggesting that innervation may persist postoperatively. However, this was not quantified, nor was the density compared to the preoperative tissue, or different regions of the capsule, healed capsule or pseudocapsule. However, the deeper layers of the pseudocapsule were found to have more FNEs than superficial layers [34]. There is no evidence to suggest that the increased density of FNEs present superior-laterally in the capsule [43, 48, 49] persists in the pseudocapsule. Further research is required to understand the changes in FNE distribution across the capsule following surgical intervention.

#### Influence of demographic variables on free nerve ending density

The influence of demographic variables has been studied in relation to FNEs density in patients with pathology, but the influence of this is unknown in healthy individuals. In the hip capsule of pathological individuals sex has no correlation with FNE density, whereas there is a significant moderate negative relationship between age and FNE density [48]; however, this may be altered according to pathological state of the joint. In patients with a fractured neck of femur some FNEs have been noted, whereas no FNEs are present in the capsules of patients with OA or pain free THA [53]. This indicates that traumatic and progressive pathology may influence FNE density differently. However, in this study the distribution of FNEs was not quantified, nor was it measured in relation to anatomical location. This therefore indicates that further study is required.

### Nerve fibres

#### Presence of nerve fibres

The current literature has investigated the presence of nerve fibres with numerous immunohistochemical markers for the general, sensory and sympathetic nerves, as well as with conventional staining. The majority of studies investigating the innervation of the capsule in pathology have noted nerve fibres, including those with CP, DDH, FNF and OA [42, 44, 45, 51–54]. Despite this, absence of nerve fibres has also been noted in DDH patients [55]. Nerve fibres have not been reported in the hip capsule of healthy individuals to date [28, 43, 46].

Sympathetic nerve fibres have been identified in the synovial tissue [45, 48, 52] and capsular ligaments [48], and also in the post-operative pseudocapsule [56]. Despite this the difference in quantity nerve expression in each structure has not been measured to date. Nor have differences been measured in relation to pain, age or sex. Concurrent to this, sensory nerves appear to be present, and these are both commonly located near blood vessels in the hip joint capsule [43, 53], and are subject to pathological changes concurrent to OA [58]. Furthermore, research indicates that there may be a relationship between sensory and sympathetic innervation of the hip synovium [52]. It is hypothesised that this balance may contribute to the pathogenesis and progression of OA [52]. However, further research is required to confirm this.

#### Pathology-dependent morphology

The morphology is variable as these nerves are found as small and large bundles [38, 39], or as single fibres throughout the capsular complex [42, 45, 54]. Research has shown both myelinated and unmyelinated nerve fibres in the capsule of individuals with joint pathology [58], yet this study did not delineate in which individuals these were noted.

The course of these fibres are undulating with a few running in a straight orientation through the capsular ligament [53]. Some authors were unable to note the type of fibre, but found both large and small fibres [54], whereas others have found α and C nerve fibres in individuals with a fractured neck of the femur [53]. The calibre of nerve fibres has not been compared between pathologies to date.

#### Distribution of nerve fibres

General agreement is present that there is increased nerve fibre density in the superior aspect of the capsule [48, 58], however the difference appears to be negligible as the distribution is also cited to be nearly homogenous throughout hip capsules [48]. Increased expression of nerve fibres has also been noted where the ligament attaches into the femur and acetabulum [48, 58]. Furthermore, nerves and small arteries and veins have been noted within a close proximity in the capsule [53, 58], suggesting a relationship may be present and thus that they may be targeted pharmacologically to manage pain or disease progression.

#### Influence of demographic variables on nerve fibre distribution

The influence of age, sex and other demographic variables on the distribution of nerve fibres requires further investigation. One study suggested that a moderate negative correlation exists between age and nerve fibre density in the capsule of those with pathology (AVN, CO, FAI, OA, SHD), this indicates a potential functional decline with increasing age [48]. This relationship has not been studied across the capsule, nor has it been measured in other pathologies nor in healthy individuals. Further to this, no relationship has been noted between nerve fibre density and sex [48].

#### Effect of pain and pathology on nerve fibres

Increased nerve fibre density may indicate a role in nociception, however research on nerve density in relation to pain in the hip capsule is inconclusive. Nerve fibres of the hip capsule appear to persist or proliferate in pathological states, as they have been found in the capsular complex of individuals with OA [44, 48, 50, 53], cerebral palsy [47, 51] and DDH [44]. Nerve expression appears to be increased in the hip capsule in relation to the sensation of pain in several pathologies, including OA [53], cerebral palsy [51] and DDH [44], suggesting a role in nociception.

Some nerve fibres appear present in the post-operative pseudocapsule of patients who have pain [34, 56, 57], while they are absent in the pseudocapsule of pain-free patients failed THA [53]. The presence of growth associated protein in the pseudocapsule indicates that proliferation of nerves may occur in association with pain [56]. This highlights a potential pharmacological target for management of pain in OA and other hip pathologies. Despite this, other investigations have failed to show a significant relationship between the density of neural tissue and pain scores [54], suggesting that other variables may affect nerve density.

Research notes different nerve expression between diseases, including an increase by around 2.5-fold in OA compared to individuals without OA [53], whereas no nerve fibres have been found in the capsule of individuals with a fractured femoral neck, which are assumed to have acute pain [50] and no long-standing disease. Significantly different expression is noted in OA, femoroacetabular impingement and avascular necrosis of the femoral head patients [48]. While others have found no difference in nerve density between fracture, idiopathic osteonecrosis, rheumatoid arthritis and OA groups [58]. This suggests that changes to density of innervation may be uniform across certain diseases, no change may occur or a change in nerve fibre density may occur due to numerous pathophysiological factors, such as disease progression and severity. In addition, there appears to be a moderate risk that this could be a false negative finding, resulting from an inappropriate methodology. The current literature indicates that the hip joint capsular complex plays a role in pain perception. However, nerve density may be a poor measure of the nociceptive role of the capsule, as it may be influenced by many variables, such as inflammatory markers [50], individual variation, as well as age [48] or sex.

## Discussion

The reviewed literature provides a comprehensive overview of the innervation of the hip capsule based on histological and immunohistochemical studies. A key finding was the inconsistency in the results and the presentation of the methodology, including the age, sex and pathology of samples and techniques employed. This is clearly represented by the variable quality of studies. At present, no reliable data is available for the human hip capsule that substantiates if, and to what extent, the capsule is innervated by the various receptors and fibre qualities. Less invasive surgical techniques in THA with preservation and repair of the capsule have proven to significantly reduce postoperative dislocation rates [7–16]. However, it is unclear if this technique can achieve further beneficial outcomes, such as better proprioception and pain relief by maintaining capsular mechanoreceptors and nerve fibres.

### Primary outcomes

Several primary outcomes have been noted from the literature, which may indicate the role of the innervation of the capsular complex.

#### 1 It is unclear if the hip capsule is an organ of proprioception and evidence towards this is inconclusive

The presence of mechanoreceptors noted in the literature [28, 43, 49, 58] suggests a potential proprioceptive role of the hip joint capsule. Further to this, the greater abundance of Pacinian corpuscles and Ruffini corpuscles [28, 49] compared to other mechanoreceptors indicates that the capsule may have a role in fast muscular response to joint movement, which is important in maintaining stability. However, in OA the combination of the decreased density of mechanoreceptors [28] and potential destructive changes to their morphology [41] may result in a reduced ability to sense and react to joint movements, a parameter that is thought to be important to prevent dislocation. The cause of changes in mechanoreceptor density remains unclear, but it may precede or be a result of the disease. Additionally, this may result in a loss of conduction of nerve signals in the latter stages of the disease, subsequently reducing potential proprioceptive function of the hip joint in OA compared to healthy individuals.

Information on mechanoreceptors lacks reliability partly indicated by the studies ‘low’ or ‘moderate’ quality and also the variety of methodologies employed. This therefore limits the conclusions that can be drawn and applied by the clinician treating the altered hip joint. Studies investigating healthy subjects appeared particularly limited, as these comprised of small sample sizes and unclear or unreliable methodologies. One study combined data from different tissue structures, therefore preventing comparison across structures and gaining specific knowledge on the hip capsule [28]. Numerous factors may explain the lack of mechanoreceptors detected using immunohistochemical methods including unsuitable stains and varying section thickness. Lack of mechanoreceptors may also result from a low level of protein present with nerve structures that is below the threshold detectable by immunohistochemical stains [53]. Additionally, failure to detect mechanoreceptors could be due to degradation of tissue, as a result of post mortem delay, omitting to measure mechanoreceptor density [50, 52, 54] or true absence of mechanoreceptors. The presence of mechanoreceptors cannot be reliably confirmed from the existing literature as immunological stains are more specific when staining nerve fibres and mechanoreceptors than using conventional histological staining methods [28, 64, 65]. Further research using these reliable methods are particularly important to determine presence of mechanoreceptors as some authors believe that proprioception is not be mediated by joint ligaments at all, and may be solely controlled by skin stretch receptors [26, 27, 66, 67], although this is also debated [68–71].

Discrepancies in the mechanoreceptor density reported between studies may have occurred as a result of the various counting methodologies employed and variable use of measures to ensure good inter- and intra-rater reliability [28, 34, 49, 50, 55]. Although the majority of papers used the Freeman and Wyke [29] classification, or modifications of this, to identify mechanoreceptors, none have stated the use of a computerised identification. As research identifying immunological markers using computerised software compared to by a trained investigator produced highly similar results, this methodology may produce more reliable results [72]. Furthermore, computerised identification may be more efficient than manual assessment, and also could help prevent oversight in detecting mechanoreceptors. Further research into the innervation of the capsule must consider all the variables affecting the reliability of the methodology. It should also investigate the changes between other pathologies, age and between sexes and substantiate if mechanoreceptors are present in the capsular complex or if joint stability is controlled by receptors in other parts of the body.

#### 2 The capsule may play a role in nociception of the hip joint, but this cannot be reliably inferred

The presence of FNEs [28, 34, 46, 48, 51, 53, 56] (which are thought to transmit nociceptive signals, alongside nerve fibres in the capsule in pathological patients [42, 47, 48, 50–55, 58]) indicates that the capsule may play a role in nociception. However, there is a debate as to whether increased nerve density is associated with pain in pathological individuals [47, 48, 51, 53, 54], or if these serve another modality. This disagreement may be due to the differences in pain intensity or demographic variables [54]. Furthermore, unsuitable methodologies may affect these results; an overestimation of density may be due to inaccurate measurement of nerve fibres in a cross section, as they have an undulating course. Overestimation may also occur from enhanced or initiation of synthesis of neural proteins in nerve fibres above the threshold that is usually detectable [53], rather than neurogenesis. Alternatively, increased neuronal sprouting may occur. Underreporting could result from inconsistent staining of nerve fibres with some immunological markers [51]. This may be a result of the loss of antigenic structure of the protein, which may occur in cadaveric tissue through decreased cell permeabilization [73]. In addition, immunohistochemical methods may be further improved; research on other joint ligaments has noted that other immunological markers may be more suitable alone or in combination, such as p75. The use of other immunological markers may increase the detection of FNEs as they may have different binding affinities [74], stain different aspects of the FNEs [75, 76] or produce more clearly stained FNEs [77]. In addition, no research has assessed the reliability of immunohistochemical markers in detecting FNEs in the hip capsule, to date. This indicates that nerve marker expression may be an invalid measure of pain, which could also be applied to inferring proprioceptive level by the density of mechanoreceptors.

Furthermore, the specific role of these nerves is unclear as few studies assess nerve fibres qualitatively [53, 54, 58]. Both α or c type fibres have been noted [53], but the relative density of each is not discussed; this is important as they have different conduction velocities and correspond to different mechanoreceptors. This information would be useful in ascertaining the proprioceptive and nociceptive function of the capsule. It should be noted that this information does not necessarily indicate the ability to transmit these signals, as it cannot be ensured that the sensory ending of these nerve fibres originates in the capsule. This is particularly important to note as these nerve fibres are predominantly undulating in their course [53], are variable [28, 43, 53], and may even affected by disease [41]. Further research is required to determine the contribution of FNEs to nociception in the hip joint, as the current literature may not reliably determine the function of the hip capsular complex, notably as these do not employ functional tests. Gaining greater understanding may be helpful in determining how they may be targeted to reduce pain.

### Secondary outcomes

Numerous secondary outcomes are highlighted from this review; these highlight potential clinical outcomes which result from greater understanding of innervation of the capsular complex.

#### 1 The superior-lateral aspect of the capsule may play a greater role than any other region in proprioception and pain perception of the hip joint

**Fig 2:**
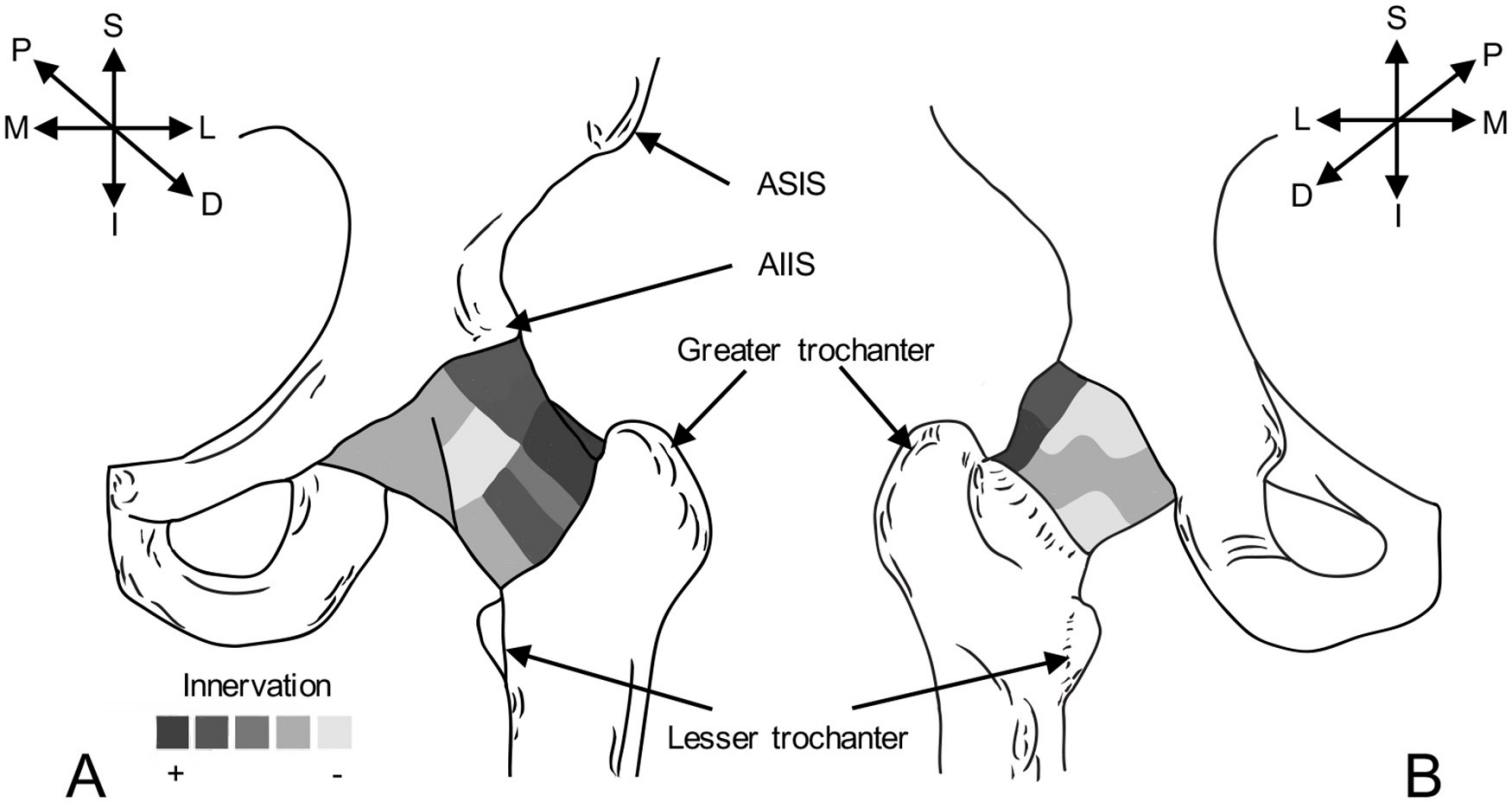
Diagram demonstrating the general agreement of innervation by mechanoreceptors I-V across the hip joint capsule. (A) Anterior view of the left side, representing increased expression laterally and superior-laterally. (B) posterior view of the left side, showing increased expression laterally. Darker regions depict areas where the literature demonstrates greater agreement of higher density of innervation. Lighter areas are regions of general agreement of little to no innervation. D = distal, I = inferior, L = lateral, M = medial, P = proximal, S = superior. ASIS = anterior superior iliac spine, AIIS = anterior inferior iliac spine.

**However, greater understanding is required in regard to the distribution of capsular innervation according to its anatomical location**. Generally, the superior-lateral aspect of the capsule [43, 48, 49], and at the attachment to bone [43, 49] has a greater density of innervation in all individuals regardless of existing pathologies (Fig 2], indicating regions of the capsule which should be avoided or handled with care during THA. However, nomenclature used to describe the parameters of the definition ‘superior’ and ‘inferior’ are inconsistent. These terms may relate to the bone or anatomical position and are not depicted diagrammatically in all studies, which make it difficult to apply these findings to the general population. Thus, further research with a distinct nomenclature is required to determine the distribution of mechanoreceptors, FNEs and nerve fibres in OA and healthy individuals. This is of clinical relevance as clinicians will attempt to preserve potential nociceptive and proprioceptive function and establish alternative surgical approaches to the hip joint, based on the anatomical descriptions of the innervation pattern. It may be hypothesized that minimal damage to capsular innervation may occur during THA with a posterior approach, with a minimally invasive incision that leaves the bony attachments of the ligament untouched. However, the literature appears inconclusive regarding which approach has better post-operative outcomes [78], yet it notes that capsular repair may be advantageous [7, 8].

Although, histological and immunohistochemical research indicates increased innervation in the superior-lateral aspect of the capsule [43, 49], a study on the gross anatomy indicates no innervation in the superior capsule between 1 o’clock and half past two (whereby the inferior acetabular notch corresponds to the 6 o’clock position) [79]. This finding may be due to an indirect origin of the nerves supplying this region, either from internally, or running through capsule from a more medial or lateral location, or due to anatomical variation. Discrepancy between these gross anatomical and histological findings may be a result of ex-vivo examination of the capsular ligaments. It is important to note that studies investigating distribution of innervation had moderate risk of bias, which was partially due to the limited methodological information provided. No study describes the methodology employed to retrieve the capsules anatomically. To simplify the interpretation, future studies should employ a clock face orientation to represent the findings, allow for comparison between studies and a possible application to clinical practice [22, 79]. Furthermore, gross anatomical study combined with immunohistochemical study may aid in understanding the relationship between density of mechanoreceptors in a region and the nerve fibres that contribute to this. This is particularly important as these nerves may be transected surgically, thus reducing the proprioceptive functioning of the hip joint. In addition, this may explain the better clinical outcomes resulting from THA with capsular repair via the posterolateral approach [7, 8], as the superior aspect of the capsule is left untouched.

#### 2 Innervation of the pseudocapsule indicates that the capsule should be preserved and repaired during total hip arthroplasty

The absence of mechanoreceptors in the pseudocapsule indicates that post-operative dislocation following THA with excision and thus without repair of the capsule may be attributed to a lack of reinnervation following the partial or complete removal of the native capsule. However, as only some individuals require revisions due to dislocation, the scarred pseudocapsule may partially contribute to the stability of the joint or some surgical approaches may preserve the innervation of the capsule. Furthermore, lower dislocation rates following THA with capsular repair [7, 8] indicates that physiological stability of the hip joint may be partially reestablished when the capsule is repaired. The current literature is unable to determine the role of innervation in the healed capsular tissue in maintaining joint stability as this has not been studied to date. In addition, the presence of a few FNEs in the pseudocapsule [34, 56] suggests that innervation may proliferate postoperatively, as supported by the presence of growth associated protein [56]. Current research is inconclusive as to what factors promote neurogenesis at the hip pseudocapsule [53]. Further research is required into pharmacological agents which may promote reinnervation of the hip capsular complex in those with capsular repair and without.

Variable factors may contribute to the reports of absence of mechanoreceptors and FNEs, including unsuitable methodologies or true absence. Furthermore, the role of the pseudocapsule cannot be inferred from the current literature as the preoperative innervation has not been studied in these individuals to date. Further investigation utilizing both histological and biomechanical investigation of proprioception may produce insightful results, as well as indicating the requirement for capsular repair.

#### 3 Surgical management strategies of the capsule in total hip arthroplasty may be dependent on age and other individual variables

*Age appears to have a significant negative relationship with nerve fibre density in the* hip capsule [48], however this is based upon a broad range of specimens with various pathologies. Despite this, research is unable to infer changes in proprioceptive or nociceptive function from nerve density in the hip capsule. Firstly, as mechanoreceptors were not described in this study [48]. Secondly, as the study omits to clearly delineate the presence of nerve fibres and FNEs [48], and the quantities of each. Thirdly, a cohort with various pathologies was studied; therefore, these findings lack specificity, as many confounding variables differed concurrently. Further investigations are required to clarify the relationship of age with innervation in healthy individuals.

Furthermore, the relationship between age and innervation may not be linear, as it could be related to the different stages of development of the hip joint. A relationship between development of the hip joint capsule and the pattern of innervation may be present [40]. Innervation of the anterior aspect of the capsule is thought to develop first, however this is based on a study with high risk of bias, an unclear description of nerve endings and with an absent methodology. It remains unknown if mechanoreceptors are present at birth [40, 46] in the human hip capsule complex, or if they appear during childhood or adolescence. The development of the capsule begins in utero [41] and the hip joint continues to develop up to the age of 35 years [80]. This indicates that the morphology of the capsule may be altered over a long period of time and therefore development may be influenced by many variables. Developmental changes have also been noted as indicators for hip joint degeneration later in life, and may also predispose the joint to denervation [81]. It is important to note numerous variables affect the progression of degeneration, as well as the density of innervation; this relationship may only be reliably studied in longitudinal studies.

Current literature lacks information on the changes in innervation according to other anthropometric and demographic variables, such as pelvic dimensions, ethnicity and sex, which may affect the density or distribution of innervation. This prevents research-informed suggestions for the surgical management of the hip joint that considers the capsule as a potential organ of proprioception. In particular, it is important to note that current studies on subjects with no known pathology have ‘moderate’ to ‘low’ risk of bias, numerous variables differ concurrently which makes it difficult to compare the findings across studies. These subjects are derived from cadaveric material donated to anatomy or pathology departments [28, 43], which tend to be from older individuals which may have a degree of pathology of the musculoskeletal system. Given that such research interests are ethically justified, ‘healthy’ cadaveric tissue could be best sampled from forensic institutes, who receive young donors with mostly traumatic causes of death.

Information on the density of innervation in young healthy subjects is lacking, suggesting that the literature may not be applicable to the greater population. One of the studies that notes mechanoreceptors in healthy individuals failed to mention the age or sex of the two donors studied [43]. While another study investigated fetuses, which were deemed as healthy, as they were terminated due to maternal factors [46]. Thus, it is unknown if these fetuses also had any developmental abnormalities, which could limit the application of these findings. Furthermore, many confounding variables are present between individuals studied, such as age and active movement, which may affect nerve density. This therefore prevents the relationship between nerve density and age from being inferred from other studies investigating OA patients, due to the lack of samples studied, abundance of individual differences and lack of reported data on the stage of disease. Although the literature indicates that age may have an effect on innervation [48], this has not been studied in a healthy cohort to date. Furthermore, this change in innervation may be concurrent to the changes in tensile properties that occur with age [22]. This indicates that surgical management may require alteration dependent on age and a different technique of capsular repair may be required in the elderly population in order to restore the proprioceptive functioning to close to its normal state. This may be necessary as in the elderly proprioceptive function is thought to be reduced as a normal part of aging. This therefore requires further study, as well as the effect of different pathologies and other demographics on innervation.

#### 4 The potential relationship between the neural and vascular systems in the hip capsular complex may highlight areas for pharmaceutical and other therapeutic interventions

A relationship may be present between the density of sympathetic and sensory nerves in the hip capsule synovium [52]. It has been hypothesised that a balance of sensory and sympathetic nerves is required for normal pain perception and tissue homeostasis to prevent joint degradation [52]. This may also play a role in the pathogenesis of degenerative joint disorders, such as OA [82]. The nerve density appears to be altered in the hip capsule synovium of patients with OA from the expected 1:1 ratio between tyrosine hydroxylase and substance P expressing nerves [52], shown to be normally exhibited in the knee joint synovium of healthy individuals [83]. However, this ratio has not been studied in the healthy hip joint to date. Furthermore, the relationship may differ according to pathology and the joint studied [52, 83, 84].

The pathogenesis of OA is complex as it involves the whole joint and many variables which may influence the density of nerves concurrent to OA progression [85]. The potential relationship between sensory and sympathetic nerves may be indirect and also regulated by numerous factors, including the immune response [84–86]. These nerves may be targeted pharmacologically through the vascular system as they are commonly noted in close proximity to blood vessels [43, 48, 58]. Further research is required to determine the sympathetic innervation of the hip capsule in healthy individuals in order to understand if there are changes in pathology and the effect of this on pain perception.

### Limitations of this review study

Few studies were noted in the literature, although a broad literature search was performed, encompassing several databases. This indicates that conclusions are based upon little comparable research, as they each employed different samples and methodologies. In addition, the literature search was performed in English, which may have excluded some relevant articles not included on these English databases, or using terms not included in the search criteria. Further to this, although the articles in languages other than English were translated by native and fluent speakers, the direct and detailed translation of these into English may have been misinterpreted. In addition, the review was not registered in PROSPERO prior to completion, as the authors were not aware of the necessity to internationally register systematic reviews when the project began. This would have, potentially, reduced the risk of reporting bias. However, it is important to note that several databases were reviewed, and papers were not excluded based on language, date, or location published, unless they lacked peer review.

## Conclusions

The current literature highlights two primary outcomes and potential roles of the innervation of the capsule: firstly, in the mechanics of the hip joint and secondly in pain perception. These roles suggest the potential for pharmaceutical and surgical interventions aiming to preserve the innervation of the capsule close to its native and unaltered state.

A coherent map of the innervation of the hip capsule with respect to anatomical location, sex, age, and pathology is lacking. Many variables differ concurrently and therefore it is difficult to compare the different studies. Increased proprioceptive and nociceptive function may be present superior-laterally compared to other regions. This indicates that lower dislocation rates and post-operative pain may result from THA which repairs the capsule in the superior-lateral region or spares this region by employing a different approach. Furthermore, less post-operative pain may result from maintaining the normal capsular tissue and its innervation. In addition, a relationship may be present between age and nerve distribution, indicating that different techniques may be required during THA in the elderly population. However, further research is required to understand post-operative innervation following THA with and without repair of the capsule. Despite the literature noting the presence of mechanoreceptors in the capsule, research employing immunohistochemical methodologies have not been successful in identifying mechanoreceptors, to date. Conventional histological stains are thought to only highlight morphologically normal mechanoreceptors, which may result in underprediction of proprioceptive function. Further research is required employing suitable immunohistochemical techniques, in order to determine the proprioceptive role of the hip capsule and its contribution to the function of the hip joint.

General agreement is present that nerve fibres and FNEs are extensive across the capsule, however their potential role in nociception requires further study. Current research highlights a potential relationship between sympathetic and sensory nerves in the hip capsular complex, which may have implications on the inflammatory response and pain perception. However, further research is required to understand the role of this in healthy and pathological individuals. This may be important for developing new pharmacological therapies for pain management and the treatment of OA.

Many individuals have OA and undergo THA each year. Minimally invasive surgical techniques during THA with capsular preservation and repair have proved to be a highly successful treatment option with reduced complication rates, including decreased dislocation rates [7–16]. It is important to ascertain a greater understanding if the distribution of innervation is crucial to its success, to aid in determining whether or not the capsule should be retained in THA. After careful review of the literature, we propose that extensive study of the distribution of innervation is required. This should include quantitative analysis of the distribution of mechanoreceptors and nerve fibres across the capsule using immunohistochemical markers, including an assessment of changes that occur with age, pathology, side and sex.

## Supporting information

S1 Table PRISMA 2009 checklist. (PDF)

## References

[1] Kurtz SM, Röder, C, Lau, E, Ong, K, Widmer, M, Maravic, M, et al. International survey of primary and revision total hip replacement. 56th Annual Meeting of the Orthopaedic Research Society. 2011;365. 10.1007/s00264-011-1235-5 PMC3224613 21404023

[2] Schwartz BE, Piponov HI, Helder CW, Mayers WF, Gonzalez MH. Revision total hip arthroplasty in the United States: national trends and in-hospital outcomes. Int Orthop. 2016;40(9):1793–802. 10.1007/s00264-016-3121-7 26830782

[3] NJR. National Joint Registry 14th Annual Report 2017. 2017.

[4] NZOA. New Zealand Joint Registry 17 year Report. 2016;0–170.

[5] Walters BL, Cooper JH, Rodriguez JA. New findings in hip capsular anatomy: dimensions of capsular thickness and pericapsular contributions. Arthroscopy. 2014;30(10):1235–45. 10.1016/j.arthro.2014.05.012 25064755

[6] Zhou Y, Cao S, Li L, Narava M, Fu Q, Qian Q. Is soft tissue repair a right choice to avoid early dislocation after THA in posterior approach? BMC Surg. 2017;17(1):60 10.1186/s12893-017-0212-3 28525999 PMC5438560

[7] Jurkutat J, Zajonz D, Sommer G, Schleifenbaum S, Mobius R, Grunert R, et al The impact of capsular repair on the risk for dislocation after revision total hip arthroplasty—a retrospective cohort-study of 259 cases. BMC Musculoskelet Disord. 2018;19(1):314 10.1186/s12891-018-2242-0 30170580 PMC6119275

[8] Prietzel T, Hammer N, Schleifenbaum S, Adler D, Pretzsch M, Kohler L, et al [The impact of capsular repair on the dislocation rate after primary total hip arthroplasty: a retrospective analysis of 1972 cases]. Z Orthop Unfall. 2014;152(2):130–43. 10.1055/s-0034-1368209 24760453

[9] Ho KW, Whitwell GS, Young SK. Reducing the rate of early primary hip dislocation by combining a change in surgical technique and an increase in femoral head diameter to 36 mm. Arch Orthop Trauma Surg. 2012;132(7):1031–6. 10.1007/s00402-012-1508-5 22460352

[10] Hummel MT, Malkani AL, Yakkanti MR, Baker DL. Decreased dislocation after revision total hip arthroplasty using larger femoral head size and posterior capsular repair. J Arthroplasty. 2009;24(6 Suppl):73–6. 10.1016/j.arth.2009.04.026 19577890

[11] White RE, Forness TJ, Allman JK, Junick DW. Effect of posterior capsular repair on early dislocation in primary total hip replacement. Clin Orthop Relat Res. 2001;393:163–7. 10.1097/00003086-200112000-00019 11764346

[12] Sierra RJ, Raposo JM, Trousdale RT, Cabanela ME. Dislocation of primary THA done through a posterolateral approach in the elderly. Clin Orthop Relat Res. 2005;441:262–7. 10.1097/01.blo.0000194308.23105.f4 16331013

[13] Iorio R, Specht LM, Healy WL, Tilzey JF, Presutti AH. The effect of EPSTR and minimal incision surgery on dislocation after THA. Clin Orthop Relat Res. 2006;447:39–42. 10.1097/01.blo.0000218750.14989.ef 16741472

[14] Tsai SJ, Wang CT, Jiang CC. The effect of posterior capsule repair upon post-operative hip dislocation following primary total hip arthroplasty. BMC Musculoskelet Disord. 2008;9:29 10.1186/1471-2474-9-29 18307820 PMC2292160

[15] Prigent F. Incidence of capsular closure and piriformis preservation on the prevention of dislocation after total hip arthroplasty through the minimal posterior approach: comparative series of 196 patients. Eur J Orthop Surg Traumatol. 2008;18(5):333–7.

[16] Khan RJ, Fick D, Khoo P, Yao F, Nivbrant B, Wood D. Less invasive total hip arthroplasty: description of a new technique. J Arthroplasty. 2006;21(7):1038–46. 10.1016/j.arth.2006.01.010 17027549

[17] Fessy MH, Putman S, Viste A, Isida R, Ramdane N, Ferreira A, et al What are the risk factors for dislocation in primary total hip arthroplasty? A multicenter case-control study of 128 unstable and 438 stable hips. Orthop Traumatol Surg Res. 2017;103(5):663–8. 10.1016/j.otsr.2017.05.014 28629944

[18] Colacchio ND, Robbins CE, Aghazadeh MS, Talmo CT, Bono JV. Total hip intraoperative femur fracture: do the design enhancements of a second-generation tapered-wedge stem reduce the incidence? J Arthroplasty. 2017;32(10):3163–8. 10.1016/j.arth.2017.05.012 28648706

[19] Hewitt J, Guilak F, Glisson R, Vail TP. Regional material properties of the human hip joint capsule ligaments. J Orthop Res. 2001;19(3):359–64. 10.1016/S0736-0266(00)00035-8 11398846

[20] Hewitt JD, Glisson RR, Guilak F, Vail TP. The mechanical properties of the human hip capsule ligaments. J Arthroplasty. 2002;17(1):82–9. 10.1054/arth.2002.27674 11805930

[21] Prietzel T, Hammer N, Schleifenbaum S, Kassebaum E, Farag M, von Salis-Soglio G. On the permanent hip-stabilizing effect of atmospheric pressure. J Biomech. 2014;47(11):2660–5. 10.1016/j.jbiomech.2014.05.013 24938930

[22] Schleifenbaum S, Prietzel T, Hadrich C, Mobius R, Sichting F, Hammer N. Tensile properties of the hip joint ligaments are largely variable and age-dependent—An in-vitro analysis in an age range of 14–93 years. J Biomech. 2016;49(14):3437–43. 10.1016/j.jbiomech.2016.09.001 27667477

[23] Pieroh P, Schneider S, Lingslebe U, Sichting F, Wolfskampf T, Josten C, et al The stress-strain data of the hip capsule ligaments are gender and side independent suggesting a smaller contribution to passive stiffness. PLoS One. 2016;11(9):e0163306 10.1371/journal.pone.0163306 27685452 PMC5042535

[24] Zijlstra WP, De Hartog B, Van Steenbergen LN, Scheurs BW, Nelissen R. Effect of femoral head size and surgical approach on risk of revision for dislocation after total hip arthroplasty. Acta Orthop. 2017;88(4):395–401. 10.1080/17453674.2017.1317515 28440704 PMC5499330

[25] Logishetty K, van Arkel RJ, Ng KCG, Muirhead-Allwood SK, Cobb JP, Jeffers JRT. Hip capsule biomechanics after arthroplasty. Bone Joint J. 2019;101-B(4):426–34. 10.1302/0301-620X.101B4.BJJ-2018-1321.R1 30929480

[26] Grigg P, Finerman G, Riley L. Joint position sense after total hip replacement. J Bone Joint Surg. 1973;55 A(5):1016–25. 4760087

[27] Dellon AL. Proprioception. In: Dellon, AL. Somatosensory testing & rehabilitation. Bethesda. American Occupational Therapy Association, Inc. 1997: 32–37. From: https://www.dellon.com/images/somatosensory/soma_section_1_cha1-4.pdf

[28] Moraes MRB, Cavalcante MLC, Leite JAD, Macedo JN, Sampaio MLB, Jamacaru VF, et al The characteristics of the mechanoreceptors of the hip with arthrosis. J Orthop Surg Res. 2011;6. 10.1186/1749-799X-6-58 PMC3233501 22087603

[29] Freeman MAR, Wyke B. The innervation of the knee joint. An anatomical and histological study in the cat. J Anat. 1967;101(3):505–32. 6051731 PMC1270929

[30] Hagert E, Garcia-Elias M, Forsgren S, Jung BO. Immunohistochemical analysis of wrist ligament innervation in relation to their structural composition. J Hand Surg Am. 2007;32a(1):30–6. 10.1016/j.jhsa.2006.10.005 17218173

[31] Tomita K, Berger EJ, Berger RA. The Discrepancy in Freeman and Wyke Classification for Joint Mechanoreceptors in Human Wrist. 52nd Annual Meeting of the Orthopaedic Research Society. 1989.

[32] Adachi N, Ochi M, Uchio Y, Iwasa J, Ryoke K, Kuriwaka M. Mechanoreceptors in the anterior cruciate ligament contribute to the joint position sense. Acta Ortho Scand. 2009;73(3):330–4. 10.1080/000164702320155356 12143983

[33] Li B, Wang Y, Bai L, Wen Y. Changes of mechanoreceptors in different-state remnants of ruptured anterior cruciate ligament. Int Orthop. 2018;42:2613–8. 10.1007/s00264-018-3933-8 29752507

[34] Naim Syed S, Phillips A, Van Pittius DG. Assessing regeneration of mechanoreceptors in human hip pseudocapsule after primary total hip arthroplasty. J Ortho Trauma Rehab. 2014;18(1):12–4.

[35] Miki H, Masuhara K. Arthrographic examination of the pseudocapsule of the hip after posterior dislocation of total hip arthroplasty. Int Ortho. 2000;24:256–9. 10.1007/s002640000166 PMC3619915 11153453

[36] Simons MJ, Amin NH, Cushner FD, Scuderi GR. Characterization of the neural anatomy in the hip joint to optimize periarticular regional anesthesia in total hip arthroplasty. J Surg Orthop Adv. 2015;24(4):221–4. 26731384

[37] Moher D, Liberati A, Tetzlaff J, Altman DG, Group P. Preferred reporting items for systematic reviews and meta-analyses: the PRISMA statement. Int J Surg. 2010;8(5):336–41. 10.1016/j.ijsu.2010.02.007 20171303

[38] Knijn N, Simmer F, Nagtegaal ID. Recommendations for reporting histopathology studies: a proposal. Virchows Arch. 2015;466(6):611–5. 10.1007/s00428-015-1762-3 25846513 PMC4460276

[39] Manterola C, Otzen T. Checklist for Reporting Results Using Observational Descriptive Studies as Research Designs: The MInCir Initiative. Int J Morphol. 2017;35(1):72–6.

[40] Golub DM, Bronovitskaia GM. [Development of the human hip joint and its innervation]. Arkh Anat Gistol Embriol. 1981;80(5):47–56. 7283755

[41] Nettov GG, Iankovskaia NF. [Reactive-destructive changes in the nervous elements of the capsule of the hip joint in coxarthrosis.]. Arkh Anat Gistol Embriol. 1978;74(2):72–4. 646640

[42] Saxler G, Löer F, von Knoch M, von Knoch F, Hanesch U. Die Lokalisation des Neurokinin 1-Rezeptors im Hüftgelenk von Patienten mit schmerzhafter Osteoarthrose. Zeitschrift für Orthopädie und ihre Grenzgebiete. 2005;143(04):424–30. 16118758 10.1055/s-2005-836832

[43] Hosokawa O. Histological study on the type and distristibution of the sensory nerve endings in the human hip joint capsule and ligament. Nippon Seikeigeka Gakkai zasshi. 1964;38:887–901. 14276180

[44] Li, Wang H, He JY, Wang CL, Feng WJ, Shen C, et al Inflammatory and fibrosis infiltration in synovium associated with the progression in developmental dysplasia of the hip. Mol Med Rep. 2019;19(4):2808–16. 10.3892/mmr.2019.9910 30720141

[45] Vasconcelos DM, Ribeiro-da-Silva M, Mateus A, Alves CJ, Machado GC, Machado-Santos J, et al Immune response and innervation signatures in aseptic hip implant loosening. J Transl Med. 2016;14(1):205 10.1186/s12967-016-0950-5 27387445 PMC4937545

[46] Desteli EE, Gulman AB, Imren Y, Kaymaz F. Comparison of mechanoreceptor quantities in hip joints of developmental dysplasia of the hip patients with normal hips. Hip Int. 2014;24(1):44–8. 10.5301/hipint.5000091 24186677

[47] Grzegorzewski A, Jóźwiak M., Pawlak M., Modrzewski T, Buchcic P., and Masłoń A. Hip joint pain in children with cerebral palsy and developmental dysplasia of the hip: why are the differences so huge? BMC Musculoskelet Disord. 2014;15(96):1–6. 24656137 10.1186/1471-2474-15-96 PMC4004466

[48] Haversath M, Hanke J, Landgraeber S, Herten M, Zilkens C, Krauspe R, et al The distribution of nociceptive innervation in the painful hip: a histological investigation. Bone Joint J. 2013;95-B(6):770–6. 10.1302/0301-620X.95B6.30262 23723270

[49] Gerhardt M, Johnson K, Atkinson R, Snow B, Shaw C, Brown A, et al Characterisation and classification of the neural anatomy in the human hip joint. Hip Int. 2012;22(1):75–81. 10.5301/HIP.2012.9042 22344482

[50] Takeshita M, Nakamura J, Ohtori S, Inoue G, Orita S, Miyagi M, et al Sensory innervation and inflammatory cytokines in hypertrophic synovia associated with pain transmission in osteoarthritis of the hip: a case-control study. Rheumatology (Oxford). 2012;51(10):1790–5. 22772321 10.1093/rheumatology/kes173

[51] Maslon A, Jozwiak M, Pawlak M, Modrzewski T, and, Grzegorzewski A. Hip joint pain in spastic dislocation: aetiological aspects. Dev Med Child Neurol. 2011;53(11):1019–23. 10.1111/j.1469-8749.2011.04077.x 21848874

[52] Lehner B, Koeck FX, Capellino S, Schubert TEO, Hofbauer R, Straub RH. Preponderance of sensory versus sympathetic nerve fibers and increased cellularity in the infrapatellar fat pad in anterior knee pain patients after primary arthroplasty. J Orthop Res. 2008;26(3):342–50. 10.1002/jor.20498 17902175

[53] Saxler G, Löer F, Skumavc M, Pförtner J, and, Hanesch U. Localization of SP- and CGRP-immunopositive nerve fibers in the hip joint of patients with painful osteoarthritis and of patients with painless failed total hip arthroplasties. Eur J Pain. 2007;11(1):67–74. 10.1016/j.ejpain.2005.12.011 16460974

[54] Gáspár L, Dezso B, Csernátony Z, Gáspár L, Szabó J, Szekanecz Z, et al Capsular neuronal elements and their relation to pain reduction and functional improvement following total hip replacement. Int Orthop. 2004;28(3):142–5. 10.1007/s00264-004-0539-0 14762693 PMC3474501

[55] Muratli HH, Bicimoglu A, Tabak YA, Celebi L, and, Paker I. Mechanoreceptor evaluation of hip joint capsule and ligamentum capitis femoris in developmental hip dysplasia: a preliminary study. J Pediatr Orthop B. 2004;13(5):299–302. 10.1097/01202412-200409000-00003 15552555

[56] Niissalo S, Li TF, Santavirta S, Takagi M, Hietanen J, Konttinen YT. Dense innervation in pseudocapsular tissue compared to aneural interface tissue in loose totally replaced hips. J Rheumatol. 2002;29(4):796–803. 11950024

[57] Bosetti M, Massè A, Navone R, Cannas M. Biochemical and histological evaluation of human synovial-like membrane around failed total hip replacement protheses during in vitro mechanical loading. J Mater Sci: Mater Med. 2001;12:693–8. 15348240 10.1023/a:1011216509099

[58] Rabinowicz T, Jacqueline F. Pathology of the Capsular and Synovial Hip Nerves in Chronic Hip Diseases. Pathol Res Pract. 1990;186(2):283–92. 10.1016/S0344-0338(11)80546-7 2343000

[59] Bombelli R, Santore RF, Poss R. Mechanics of the normal and osteoarthritic hip. A new perspective. Clin Orthop Relat Res. 1984;182:69–78. 6692629

[60] Crowe J, Mani V, Ranawat C. Total hip replacement in congenital dislocation and dysplasoa of the hip. J Bone Joint Surg. 1979;61(1):15–23. 365863

[61] Harris W. Traumatic arthritis of the hip after dislocation and acetabular fractures: treatment by mold arthroplasty. An end result study using a new method of result evaluation. J Bone Joint Surg. 1969;51(4):737–55. 5783851

[62] Kellgren JH, Lawrence JS. Radiological Assessment of Osteo-arthrosis. Ann Rheum Dis. 1957;16:494–502. 10.1136/ard.16.4.494 13498604 PMC1006995

[63] Busse J, Gasteiger W, Tonnis D. [A new method for roentgenologic evaluation of the hip joint-the hip factor]. Arch Orthop Unfallchir. 1971;72:1–9. 10.1007/BF00415854 5020681

[64] Dhillon MS, Bali K., and, Prabhakar S. Differences among mechanoreceptors in healthy and injured anterior cruciate ligaments and their clinical importance. Muscles, Ligaments and Tendons J. 2012;2(1):38–43. 23738272 PMC3666492

[65] Bali K, Dhillon MS, Vasistha RK, Kakkar N, Chana R, Prabhakar S. Efficacy of immunohistological methods in detecting functionally viable mechanoreceptors in the remnant stumps of injured anterior cruciate ligaments and its clinical importance. Knee Surg Sports Traumatol Arthrosc. 2012;20(1):75–80. 10.1007/s00167-011-1526-9 21541706

[66] Moberg E. The role of cutaneous afferents in position sense, kinaesthesia, and motor function of the hand. Brain. 1983;106:1–19. 10.1093/brain/106.1.1 6831192

[67] Dellon AL. Commentary: Desensitizing the posterior interosseous nerve alters wrist proprioceptive reflexes: it is ok to lose your nerve. J Hand Surg Am. 2010;35(7):1067–9. 10.1016/j.jhsa.2010.04.027 20610050

[68] Hagert E. Comment to "Desensitizing the posterior interosseous nerve alters wrist proprioceptive reflexes". J Hand Surg Am. 2010;35(12):2131–2. 10.1016/j.jhsa.2010.09.035 21074950

[69] Hagert E, Persson JK. Desensitizing the posterior interosseous nerve alters wrist proprioceptive reflexes. J Hand Surg Am. 2010;35(7):1059–66. 10.1016/j.jhsa.2010.03.031 20610049

[70] Reimann BL, Lephart SM. The sensorimotor system, part I: the physiologic basis of functional joint stability. J Athl Training. 2002;37(1):71–9. PMC164311 16558670

[71] Reimann BL, Lephart SM. The sensorimotor system, part II: the role of proprioception in motor control and functional joint stability. J Athl Training. 2002;37(1):80–4. PMC164312 16558671

[72] Bankhead P, Fernandez JA, McArt DG, Boyle DP, Li G. Integrated tumor identification and automated scoring minimizes pathologist involvement and provides new insights to key biomarkers in breast cancer. Lab Invest. 2018;98(1):15–26. 10.1038/labinvest.2017.131 29251737

[73] Beckmann J, Knodl M, Bauser E, Tingart M, Grifka J, Straub RH. Loss of sympathetic nerve fibers in vital intertrochanteric bone cylinders lateral to osteonecrosis of the femoral head. Joint Bone Spine. 2013;80(2):188–94. 10.1016/j.jbspin.2012.03.003 22575068

[74] Leunig M, Beck M., Stauffer E., Hertel R. and Ganz R. Free nerve endings in the ligamentum capitis femoris. Acta Anaesthesiol Scand. 2000;71(5):452–4. 10.1080/000164700317381117 11186399

[75] Hagert E, Lluch A, Rein S. The role of proprioception and neuromuscular stability in carpal instabilities. J Hand Surg Eur Vol. 2015;41(1):94–101. 10.1177/1753193415590390 26115684

[76] Rein S, Hanisch U, Zwipp H, Fieguth A, Lwowski S, Hagert E. Comparative analysis of inter- and intraligamentous distribution of sensory nerve endings in ankle ligaments: a cadaver study. Foot Ankle Int. 2013;34(7):1017–24. 10.1177/1071100713480862 23456084

[77] Sha L, Xie G, Zhao S, Zhao J. A morphologic and quantitative comparison of mechanoreceptors in the tibial remnants of the ruptured human anterior cruciate ligament. Medicine (Baltimore). 2017;96(5):e6081. 28151920 10.1097/MD.0000000000006081 PMC5293483

[78] Onishi H, Nagoya S, Takebayashi T, Yamashita T. Analysis of Proprioception of Hip Joint in Total Hip Arthroplasty. Open Orthop J. 2017;07(02):53–62.

[79] Kampa RJ, Prasthofer A, Lawrence-Watt DJ, Pattison RM. The internervous safe zone for incision of the capsule of the hip. A cadaver study. J Bone Joint Surg Br. 2007;89(7):971–6. 10.1302/0301-620X.89B7.19053 17673597

[80] Scheuer L, Black S. The Juvenile Skeleton. London: Elsevier Academic Press; 2004.

[81] Vanden Berg-Foels WS, Schwager SJ, Todhunter RJ, Reeves AP. Femoral head shape differences during development may identify hips at risk of degeneration. Ann Biomed Eng. 2011;39(12):2955–63. 10.1007/s10439-011-0393-3 21909817

[82] Grässel SG. The role of peripheral nerve fibers and their neurotransmitters in cartilage and bone physiology and pathophysiology. Arthritis Res Ther. 2014;16(6):485 10.1186/s13075-014-0485-1 25789373 PMC4395972

[83] Weidler C. Low density of sympathetic nerve fibres and increased density of brain derived neurotrophic factor positive cells in RA synovium. Ann Rheum Dis. 2005;64(1):13–20. 10.1136/ard.2003.016154 15608299 PMC1755208

[84] Capellino S, Weber K, Gelder M, Harle P, Straub RH. First appearance and location of catecholaminergic cells during experimental arthritis and elimination by chemical sympathectomy. Arthritis Rheum. 2012;64(4):1110–8. 10.1002/art.33431 22034154

[85] Man GS, Mologhianu G. Osteoarthritis pathogenesis–a complex process that involves the entire joint. J Med Life. 2014;7(1):37–41. 24653755 PMC3956093

[86] Ribeiro-da-Silva M, Vasconcelos DM, Alencastre IS, Oliveira MJ, Linhares D, Neves N, et al Interplay between sympathetic nervous system and inflammation in aseptic loosening of hip joint replacement. Sci Rep. 2018;8(1):16044 10.1038/s41598-018-33360-8 30375409 PMC6207762

